# Supplementary material for: Pathologic Changes in and Immunophenotyping of Polymyositis in the Dutch Kooiker Dog
Source: Animals (Basel). 2024 Aug 29;14(17):2519. doi: 10.3390/ani14172519 (PMC11394232; doi:10.3390/ani14172519)
Supplement: Supplementary file 1 [file animals-14-02519-s001.zip › animals-3108509-supplementary.pdf]

animals

MDPI

Supplementary materials:
Table S1: Description and scoring of the histological variables.

Histologic Parameter	Definition	Scoring Method[1]/Grading[2]
Distribution of Infiltrates	Distribution of Infiltrates within the muscle section	
Fiber directed	Inflammatory cells invading non-degenerated myofibres	0: Absent<br>1: Present
Interstitial	Inflammatory infiltrates scattered in the endomysium/perimysium	0: Absent<br>1: Present
Coring out of fibers	Inflammatory cells infiltrates the central part of the myofibre	0: Absent<br>1: Present
Within the fascia	Inflammatory infiltrates within the fascia	0: Absent<br>1: Present
Diffuse	Inflammatory infiltrates scattered diffusely throughout the whole muscle fascicle	0: Absent<br>1: Present
Random	Inflammatory infiltrates scattered randomly without identifiable pattern	0: Absent<br>1: Present
Perivascular	Inflammatory infiltrates cluster around blood vessels	0: Absent<br>1: Present
Intramural of blood vessels	Inflammatory infiltrates within the blood vessel wall	0: Absent<br>1: Present
Leukostasis	Intravascular accumulation of white blood cells	0: Absent<br>1: Present
Pattern of infiltration within the fascicle	Pattern of inflammatory infiltration within the affected fascicle	
Peripheral	Infiltration is more severe in the periphery and spreads towards the centre	0: Absent<br>1: Present
Central	Infiltration is more severe in the centre and spreads towards the periphery	0: Absent<br>1: Present
Diffuse	Diffuse infiltration throughout the whole fascicle	0: Absent<br>1: Present
Random	Random infiltration without identifiable pattern	0: Absent<br>1: Present
Myofiber Pathology		
Diameter variation	Size variation of the myofibres	0: Absent<br>1: Mild<br>2: Moderate<br>3: Severe
Polygonal atrophy	Atrophic myofibres, with polygonal/rounded outlines	0: Absent<br>1: Mild<br>2: Moderate<br>3: Severe

Angular atrophyc	Atrophic myofibres, with outlines forming acute angles	0: Absent<br>1: Mild<br>2: Moderate<br>3: Severe
Hypertrophic	Increased myofibre size	0: Absent<br>1: Mild<br>2: Moderate<br>3: Severe
Nuclear position	Variations of nuclear position within the sarcoplasm	1: normal<br>2: abnormal; nuclear internalisation
Myofiber splitting	Hypertrophic fibre with a clear cleavage dividing the myofibre in 2	0: Absent<br>1: Present
Mineralisation	Calcium accumulation within muscle fibres	0: Absent<br>1: Present
Myofiber necrosis	Coagulative necrosis, from early stage (Zenker's degeneration) to macrophages invasion.	0: Absent<br>1: Present
-if present: mono- or polyphasic	Monophasic: necrotic myofibres are all at the same stage.<br>Polyphasic: necrotic myofibres are at different stages	0: Absent<br>1: Present
Myofiber regeneration	Myofibres smaller in size with enlarged and central myonuclei, basophilic cytoplasm	0: Absent<br>1: Present
Stroma		
Expanded/fibrosis	Increased amount of fibrocollagenous tissue within the fascicle	0: Absent<br>1: Mild<br>2: Moderate<br>3: Severe
Lipomatosis	Increased amount of adipose tissue within the fascicle	0: Absent<br>1: Mild<br>2: Moderate<br>3: Severe
Intramuscular Nerve Branches		0: Not Seen<br>1: Seen
Vasculitis	Infiltration of inflammatory cells within the vessel wall	0: Absent<br>1: Present
Grade	Severity of myositis	1: mild<br>2: moderate<br>3: severe
Specific inflammatory cells	Semiquantitative evaluation of inflammatory cell types expressed as estimated percentage on the total population of inflammatory cells in the section.	

Neutrophils		0: Absent<br>1: <25%<br>2: 26 – 50%<br>3: 51 - 75%<br>4: >75%
Plasma Cells		0: Absent<br>1: <25%<br>2: 26 – 50%<br>3: 51 - 75%<br>4: >75%
Eosinophils		0: Absent<br>1: <25%<br>2: 26 – 50%<br>3: 51 - 75%<br>4: >75%
Mast cells		0: Absent<br>1: <25%<br>2: 26 – 50%<br>3: 51 - 75%<br>4: >75%
CD3 positive T-cells		0: Absent<br>1: <25%<br>2: 26 – 50%<br>3: 51 - 75%<br>4: >75%
CD8 positive T-cells		0: Absent<br>1: <25%<br>2: 26 – 50%<br>3: 51 - 75%<br>4: >75%
CD20 positive B-cells		0: Absent<br>1: <25%<br>2: 26 – 50%<br>3: 51 - 75%<br>4: >75%
IBA-1 positive macrophages		0: Absent<br>1: <25%<br>2: 26 – 50%<br>3: 51 - 75%<br>4: >75%
CD4	Severity of CD4+ cell infiltration and characterization of infiltrative pattern	0: absent<br>1: mild

		2: moderate<br>3: severe
Distribution of inflammatory cell types within the fascicles		
Fiber directed	Inflammatory cells invading non-degenerated myofibres	0: Absent<br>1: Present
Interstitial	Inflammatory infiltrates scattered in the endomysium/perimysium	0: Absent<br>1: Present
Within the fascia	Inflammatory infiltrates within the fascia	0: Absent<br>1: Present
Perivascular	Inflammatory infiltrates cluster around blood vessels	0: Absent<br>1: Present
Intramural of blood vessels	Inflammatory infiltrates within the blood vessel wall	0: Absent<br>1: Present
Random	Inflammatory infiltrates scattered randomly without identifiable pattern	0: Absent<br>1: Present
Sarcolemmal MHC-II	Semiquantitative evaluation expressed as percentage of positive myofibres on the total myofibres featured	0: Absent<br>1: <25%<br>2: 26 – 50%<br>3: 51 - 75%<br>4: >75%

[1]Scoring of severity:
non-significant: 0-5% of tissue affected;
mild: 5-25% of tissue affected;
moderate: 26-50% of tissue affected;
severe: >51 % of tissue affected

[2]Grading of semiquantitative evaluation of inflammatory cells:
grade 0 no staining;
grade 1 staining of 1 - 25% of the total inflammatory cell population;
grade 2 staining 26 - 50% of the total inflammatory cell population;
grade 3 staining of 51 - 75% of total inflammatory cell population;
grade 4 staining of 76 - 100% of the total inflammatory cell population.

Supplementary materials:

Table S2: Histological parameters, units and detailed number of cases

Histologic Parameter	Unit	Number of cases	(%)
Signalement			
Age	Puppy (>1y)	0	0%
	Young dog (1 – 2y)	8	21%
	Adult (2 – 10y)	31	79%
	Geriatric (<10y)	0	0%
	Unknown	0	0%
Gender	Female	12	31%
	Female spayed	7	18%
	Male	15	38%
	Male neutered	5	13%
	Unknown	0	0%
Distribution of Infiltrates			
Fiber directed	present	39	100%
Interstitial	present	39	100%
Coring out of fibers	present	24	62%
Within the fascia	present	4	10%
Diffuse	present	4	10%
Random	present	0	0%
Perivascular	present	0	0%
Intramural of blood vessels	present	0	0%
Leukostasis	present	15	38%
Pattern of infiltration within the fascicle			
Peripheral	present	12	31%
Central	present	1	3%
Diffuse	present	6	15%
Random	present	20	51%
Myofiber Pathology			
Diameter variation	absent	0	0%
	mild	19	49%
	moderate	18	46%
	severe	2	5%
Polygonal atrophy	absent	0	0%
	mild	11	28%
	moderate	23	59%
	severe	5	13%
Angular atrophy	absent	32	82%
	mild	7	18%
Hypertrophic	absent	29	74%
	mild	7	18%
	moderate	3	8%

Nuclear position	normal	39	100%
	abnormal	0	0%
Myofiber splitting	present	2	5%
Mineralization	present	1	3%
Myofiber necrosis	present	39	100%
	monophasic	0	0%
	polyphasic	39	100%
Myofiber regeneration	present	21	24%
Stroma			
Expanded/fibrosis	absent	20	51%
	mild	11	28%
	moderate	8	21%
	severe	0	0%
Lipomatosis	absent	17	44%
	mild	11	28%
	moderate	9	23%
	severe	2	5%
Intramuscular Nerve Branches	not seen	13	33%
	seen, normal	25	64%
	inflammation	1	3%
Vasculitis	present	0	0%
Grade	mild	8	21%
	moderate	17	44%
	severe	14	36%
Specific inflammatory cells			
Neutrophils	absent	10	26%
	1 - 25%	28	72%
	26 - 50%	1	3%
	51 - 75%	0	0%
	76 – 100%	0	0%
	Fiber Directed	12	41%
	Interstitial	27	93%
	Within the fascia	3	10%
	Perivascular	9	31%
	Intramural of blood vessels	0	0%
	Random	0	0%
Plasma cells	absent	10	26%
	1 - 25%	28	72%
	26 - 50%	1	3%
	51 - 75%	0	0%
	76 – 100%	0	0%
	Fiber Directed	12	41%

	Interstitial	27	93%
	Within the fascia	3	10%
	Perivascular	9	31%
	Intramural of blood vessels	0	0%
	Random	0	0%
Eosinophils	absent	28	72%
	1 - 25%	11	28%
	26 - 50%	0	0%
	51 - 75%	0	0%
	76 – 100%	0	0%
	Fiber Directed	10	91%
	Interstitial	7	64%
	Within the fascia	0	0%
	Perivascular	0	0%
	Intramural of blood vessels	0	0%
	Random	0	0%
Mast cells	absent	2	5%
	1 - 25%	32	82%
	26 - 50%	5	13%
	51 - 75%	0	0%
	76 – 100%	0	0%
	Fiber Directed	1	3%
	Interstitial	37	100%
	Within the fascia	22	59%
	Perivascular	31	84%
	Intramural of blood vessels	0	0%
	Random	1	3%
CD3 positive T-cells	absent	1	3%
	1 - 25%	6	15%
	26 - 50%	22	56%
	51 - 75%	10	26%
	76 – 100%	0	0%
	Fiber Directed	38	100%
	Interstitial	37	97%
	Within the fascia	4	11%
	Perivascular	1	3%
	Intramural of blood vessels	0	0%
	Random	0	0%
CD8 positive T-cells	absent	15	38%
	1 - 25%	21	54%
	26 - 50%	3	7%
	51 - 75%	0	0%

	76 – 100%	0	0%
	Fiber Directed	24	100%
	Interstitial	22	92%
	Within the fascia	0	0%
	Perivascular	0	0%
	Intramural of blood vessels	0	0%
	Random	0	0%
CD20 positive T-cells	absent	0	0%
	1 - 25%	35	90%
	26 - 50%	4	10%
	51 - 75%	0	0%
	76 – 100%	0	0%
	Fiber Directed	14	36%
	Interstitial	39	100%
	Within the fascia	0	0%
	Perivascular	3	8%
	Intramural of blood vessels	0	0%
	Random	0	0%
IBA-1 positive macrophages	absent	1	3%
	1 - 25%	1	3%
	26 - 50%	34	87%
	51 - 75%	3	7%
	76 – 100%	0	0%
	Fiber Directed	37	97%
	Interstitial	37	97%
	Within the fascia	1	3%
	Perivascular	0	0%
	Intramural of blood vessels	0	0%
	Random	0	0%
Sarcolemmal MHC-II	no	5	13%
	1 - 25%	15	38%
	26 - 50%	15	38%
	51 - 75%	4	10%
	76 - 100%	0	0%
